# Intermittent Use of Continuous Glucose Monitoring in Type 2 Diabetes Is Preferred: A Qualitative Study of Patients’ Experiences

**DOI:** 10.1177/26350106251326517

**Published:** 2025-03-21

**Authors:** Bente Elisabeth Bendixen, Ane Wilhelmsen-Langeland, Kirsten Lomborg, Eirin Måkestad, Marjolein M. Iversen, Eirik Søfteland, Anne Haugstvedt

**Affiliations:** Department of Health and Caring Sciences, Western Norway University of Applied Sciences, Bergen, Hordaland, Norway; Department of Health and Caring Sciences, Western Norway University of Applied Sciences, Bergen, Hordaland, Norway; Division of Psychiatry, Haukeland University Hospital, Bergen, Norway; Department of Health and Caring Sciences, Western Norway University of Applied Sciences, Bergen, Hordaland, Norway; Copenhagen University Hospital - Steno Diabetes Center Copenhagen, Copenhagen, Denmark; Department of Clinical Medicine, University of Copenhagen, Copenhagen, Denmark; Department of Medicine, Haukeland University Hospital, Bergen, Norway; Department of Health and Caring Sciences, Western Norway University of Applied Sciences, Bergen, Hordaland, Norway; Department of Medicine, Haukeland University Hospital, Bergen, Norway; Department of Health and Caring Sciences, Western Norway University of Applied Sciences, Bergen, Hordaland, Norway

## Abstract

**Purpose:**

The purpose of the study was to explore experiences with use of a continuous glucose monitor (CGM) in people with type 2 diabetes (T2DM).

**Methods:**

A qualitative study with individual semistructured interviews at 2 time points was conducted; first with 14 adults, ages 45 to 74 years (8 women) and second with 9 of the first interviewed adults (5 women) approximately 2 years later. Participants used CGM before, during, and 3 months after the concentrated group intervention. Thematic analysis was performed on the transcribed interviews.

**Results:**

Three main themes were identified regarding the use of CGMs: (1) a gamechanger in diabetes education, (2) intermittent use is preferred, and (3) a balancing act. The participants described the use of CGMs as a valuable tool in diabetes education. It increased their understanding of insulin demand and sensitivity and strengthened their awareness of how to make more health-promoting micro-choices in everyday life. Intermittent use was described as the preferred way of using CGMs. Some experienced that CGMs could be challenging, and in periods of satisfactory glucose control, CGM use was experienced as unnecessary, underpinning intermittent use as appropriate.

**Conclusion:**

Study findings showed that participants with T2DM experienced CGMs as a valuable tool to gain deeper understanding of processes in the body, which could improve diabetes self-management. CGMs can facilitate more healthy micro-choices in life. Intermittent use of CGMs is most often the preferred approach for people with T2DM, but access to CGMs should take individual preferences into consideration.

The global increase in type 2 diabetes (T2DM) presents challenges both for individuals living with the disease and for health care systems.^1^ The treatment of T2DM targets multiple clinical outcomes, and an increasing number of medical approaches are showing promising results.^2^ In addition, T2DM involves lifestyle-related aspects that demand attention. Consequently, T2DM self-management often involves various complex considerations and choices in a person’s everyday life. In clinical follow-up, individual treatment and goal setting is recommended. Diabetes self-management education (DSME) is generally regarded as a crucial component and should include support of self-reflection and practical handling with the aim to decrease the risk of adverse outcomes.^3,4^

The ability to manage glucose regulation is one of the essential tasks in T2DM, and studies have shown that a high degree of glucose variability (ie, fluctuations of glucose over a given time) is correlated with increased risk of microvascular and macrovascular complications and all-cause mortality.^5^ The daily self-management can be perceived as challenging, and it may entail psychological distress impacting overall health and quality of life.^6^ The ability to understand and use health information to make informed decisions about one’s own health is defined as health literacy.^7^ Studies indicate that insufficient health literacy can hinder effective self-management and glycemic control in people with T2DM.^8-10^

The numerous daily choices related to T2DM also require knowledge about how insulin works in the body and how various actions related to food and activity influence the effect of insulin and insulin sensitivity. To gain such knowledge, self-monitoring of blood glucose (SMBG) is essential. In T2DM, finger-prick SMBG is the most common method, and decisions about medication, diet, and activity may be influenced and motivated by these measurements. However, for SMBG to have an impact on blood glucose regulation, it requires a high number of well-structured measurements.^11,12^ A continuous glucose monitor (CGM) measures the interstitial glucose levels and provides visual data (trends and summaries) that may lead to improved understanding and self-management.^13^ The use of CGMs has been demonstrated to be beneficial for people with type 1 diabetes on several measurable outcomes.^14^ Its importance in T2DM is still debated, but growing evidence points to the value of CGMs also for this group.^14-18^ However, few studies are available on how people with T2DM experience the use of CGMs. Thus, the purpose of this study was to explore experiences with use of CGMs in people with T2DM who participated in an interdisciplinary concentrated group intervention.

## Methods

### Design

The study employed a qualitative, longitudinal descriptive design, involving individual interviews at 2 time points following 3 14-day periods with CGMs, all conducted as a part of a concentrated group intervention for people with T2DM. The participants used CGMs as an add-on learning tool before, during and after intervention. Results from the concentrated intervention have been published elsewhere.^19^ The timing of interviews was selected to gain maximum insight into participants’ perceptions of CGMs both shortly after the use of CGMs and 2 years later. This gave insight into how experiences and beliefs evolved over time.^20^

### CGM as Add-on to a Group-Based Intervention

The micro-choice-based, concentrated group intervention was a transdiagnostic intervention delivered by Helse i Hardanger in Western Norway.^21^ The intervention was originally developed for various psychological disorders^22-25^ and was adapted to a wide range of chronic diseases, including T2DM.^21^ The T2DM-adapted intervention took place over 4 days and included an interdisciplinary team consisting of an endocrinologist, a psychologist, diabetes nurses, a pharmacist, a nutritionist, and a physiotherapist. The cornerstones of the intervention were (a) preparation through 2 to 3 telephone calls and a preparation meeting normally 2 weeks before the on-site intervention, (b) focusing on the many daily micro-choices everybody makes and how the choices may enhance health, and (c) facilitating integration of the changes into everyday life after the intervention. Focusing on the many micro-choices represents a shift away from exclusively targeting glucose levels and toward examining how specific choices can improve the utilization of insulin (self-produced or injected). The approach was favorably received by the participants.^26^

CGM use was used as an “add-on” intervention to support the participant’s individual choices and behavior change projects. CGMs are not commonly used among people with T2DM in Norway, and the participants in this study had not used CGMs prior to attending the concentrated group intervention. During the intervention, they used CGMs (FreeStyle Libre 2; Abbott Diabetes Care, Alameda, California, USA) for three 14-day periods: (1) during the preparation phase 2 to 4 weeks prior to the intervention, (2) during and 1 week after the intervention, and (3) 3 months after the intervention. Instructions were given on how to use the CGM device and how to scan the device every eighth hour to ensure continuous measurement.

### Participants

Study participants were recruited purposively to secure a broad range of experiences.^27^ People with T2DM who had participated in the intervention during spring and autumn 2021 and who were cluster-randomized to use CGMs during the intervention were invited to participate in this qualitative study. Participants included were on various types of diabetes treatment regimens (insulin treatment, treatment with other glucose-lowering medications than insulin, or a combination between insulin and other medications) and had varying degrees of diabetes-related complications and comorbidities.

The interviews took place at 2 time points. For the first interviews, a diabetes nurse contacted eligible participants by phone. The nurse provided additional information about the study and received verbal approval for the researcher to contact them. Then the interviewer (BEB) contacted the participants and made appointments for the interviews. In total, 14 of the invited 15 participants consented, 8 women and 6 men, ages 45 to 74 years (Table 1).

**Table 1.** Participants and Settings^a^

First interview: 3 mo after the intervention (fall 2021/spring 2022)			Second interview: 2 y after the intervention (fall 2023)		
Participant	Diabetes duration (y)	Setting	Participant	Setting	Use of CGM after the intervention (yes/no)
1 (F)	3	Telephone	X	Telephone	No
2 (M)	13	Café	X	Participant’s home	No
3 (F)	10	Participant’s home	—	—	—
4 (M)	6	Telephone	—	—	—
5 (F)	12	Telephone	X	Telephone	Yes (continuously)
6 (F)	24	Telephone	X	Café	No
7 (F)	4	Telephone	—	—	—
8 (F)	5	Telephone	—	—	—
9 (M)	1	Telephone	X	Telephone	No
10 (M)	18	Telephone	X	Telephone	Yes (intermittent)
11 (F)	15	Telephone	X	Telephone	Yes (intermittent)
12 (F)	19	Telephone	X	Telephone	No
13 (M)	5	Telephone	X	Telephone	No
14 (M)	16	Telephone	—	—	—

Abbreviations: CGM, continuous glucose monitor; F, female; M, male.

a Behind each quote in the text, each participant is referred to with participant number and “first” or “second” to indicate interview time point.

For the second interviews, the same participants were contacted. Written and oral information was given, and 9 of the 14 participants consented to participate: 5 women and 4 men, ages 45 to 74 years (Table 1).

### Data Collection

Interview guides were developed by members of the research team (BEB, AH, AWL, KL) for both the first and the second interviews. The questions focused on the participants’ experiences with using CGMs before, during, and after participation in the concentrated intervention. The interviews started with an opening question about the participants and their diabetes to make them familiar with the interview situation before addressing main topics. Examples of main topics were (a) overall experiences with the use of CGMs, (b) value of CGMs, (c) if use of CGMs resulted in new knowledge, and (d) if CGMs contributed to changes related to nutrition and activity. Appropriate probes were determined in the situation, and some were prepared in advance.

One pilot interview was conducted prior to both interview time points. These resulted in only minor adjustments of the interview guide and were therefore included in the study. The participants were given a choice of conducting the interviews face-to-face or by telephone. In both interview situations, only 2 participants chose to meet face-to-face (Table 1). The first interviews lasted between 20 and 41 minutes, and the follow-up interviews lasted between 10 and 43 minutes. All interviews were audiotaped and transcribed verbatim.

### Data Analysis

**Figure 1.**
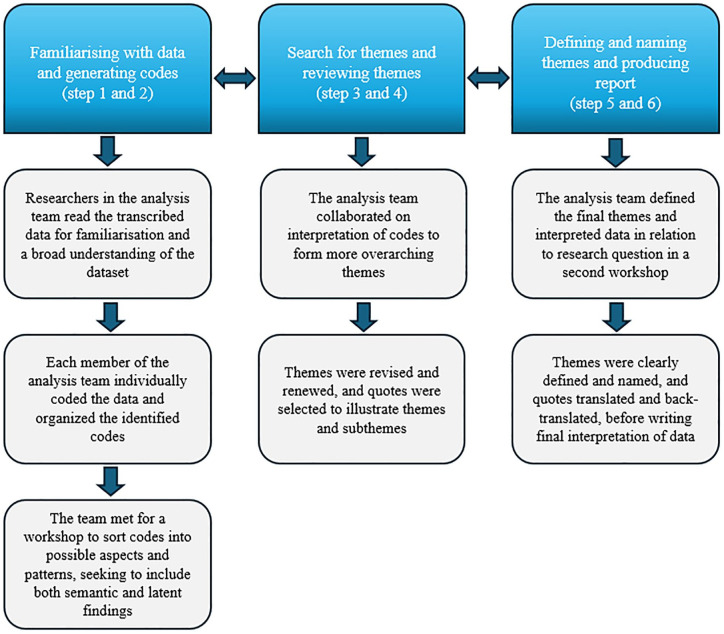
Analysis process.

The data analysis followed the 6 steps of reflexive thematic analysis as described by Braun and Clarke.^28^ The analysis process is illustrated in Figure 1. The analysis team consisted of female researchers (registered nurses, BEB, AH, EM; psychologist, AWL), all with extensive diabetes knowledge and firsthand knowledge of providing diabetes care in primary or specialist health care services. Additionally, BEB, AH, and AWL have broad experience in diabetes research and conducting qualitative studies. Steps to enhance trustworthiness and credibility were taken throughout the analysis, such as workshops for the analysis team to collaborate on creating common codes and themes. Reflexivity was enhanced by involving multiple researchers with various professional backgrounds in the analysis team. Reflexivity was further enhanced by overt awareness of the researchers’ background and beliefs throughout the analysis process. During the analysis process, quotes were used to ensure that identified themes emerged from the data rather than from the researchers’ preconceptions. To further enhance understanding and reflexivity, themes and subthemes were then presented to the remaining members of the project team (endocrinologist, ES; professors in person-centered diabetes care, KL, MI) for further collaboration and agreement on results and finally, drafting of manuscript.

### Ethics

Before the participants were included in the concentrated group intervention, they received written information about the possibility to be contacted for inclusion in various follow-up studies, including the cluster randomized controlled trial and qualitative interview studies. All participants gave written consent to participate in both interviews. Furthermore, before both interviews, the participants were reinformed that participation was voluntary and that they could withdraw from the study at any time. The informed consent included information about publication of anonymized responses. The interviewer (BEB) did not have any relationship with the participants before the first interviews. The data were de-identified. The study was conducted in accordance with the Declaration of Helsinki and approved by the Reginal Ethics Committee for Human Research in the Western region (REK Vest 2020-101648 and 2020-203941).

## Results

Three main themes about the participants’ experiences with the use of CGMs were identified: a gamechanger in diabetes education, intermittent use is preferred, and a balancing act. Under each theme, subthemes were identified, as shown in Table 2. In the following section, themes and subthemes are presented in text and with quotes.

**Table 2.** Themes and Subthemes

Themes					
A game changer in diabetes education		Intermittent use is preferred		A balancing act	
Subthemes, first interview	Subthemes, follow-up interview	Subthemes, first interview	Subthemes, follow-up interview	Subthemes, first interview	Subthemes, follow-up interview
New understanding of glucose variability Motivating and awareness-rising Improved self-management	A valuable learning tool Improved self-management is still present	Access to continuous glucose monitor is considered crucial	Useful on special occasions to gain overview No need to measure continuously	Focus on glucose level can be stressful Individual preferences is important	Continuous use can increase negative feelings Perceived control causes less need for measurements

### A Gamechanger in Diabetes Education

Initially, participants with T2DM described the use of CGMs as a revolutionary experience. The ability to monitor their glucose levels at any given moment was described as remarkable. The use of CGMs enhanced their understanding of the physiological responses to various activities and actions, and one said:

It was exceptional that you can just swipe and then you immediately have it [the glucose level]. And if you feel unwell or tired you can see how it is and what you have to do. At the second you need it, and wherever you are. . . . It is fantastic . . . , a new way of living. (Informant [Inf] 3, first interview [int])

The participants experienced the use of CGMs as motivating because it enabled them to have a more conscious and enlightened reflection about their T2DM and their diabetes self-management. The CGM was perceived as a learning tool that was fruitful in combination with the education received from the interdisciplinary team during the concentrated intervention. One participant said:

I can say that in the fifteen years before I used CGM, I didn’t understand my diabetes. I have without doubt learned a lot. (Inf 14, first int)

The periods with CGMs were actively used to understand which food resulted in a rapid rise in glucose level and which gave a less rapid rise. Furthermore, the knowledge regarding the impact of various activities on the body’s insulin need was highlighted. Participants appreciated being able to visually observe the effects of a short walk or certain foods on glucose levels. One said:

And we had lunch during the treatment program, and we went for a little walk, just fifteen minutes . . . and we could see the effect on the glucose level immediately, which was surprising. (Inf 9, first int)

The concentrated intervention focused on the many micro-choices one makes during a day. The use of CGMs gave the participants an increased understanding of how these various micro-choices impact the body’s need for insulin or the body’s insulin sensitivity. One commented:

I would never have thought that it actually has such a big effect; the little things that I can do or eat. That it has such a big impact. . . . So now I haven’t used my car for work at all [after using the CGM]. I have walked, because it has such a positive effect on my glucose levels. (Inf 12, first int)

Some participants described that they usually did not have any symptoms due to high or low glucose levels and therefore were unable to act on it. When using CGMs, they understood the body’s signals when glucose levels were out of range and could initiate actions immediately to enhance the effect of the insulins (ie, go for a walk) or action to avoid a further lowering of the glucose level. The follow-up interviews revealed that even without CGMs, this new ability to recognize symptoms was still present 2 years after the intervention. One commented:

I learned [by using the CGM] that I can go very low [glucose level], and I was not aware of that, because I didn’t feel anything. . . . So now I am aware of which symptoms I have when my glucose level is low. (Inf 1, second int)

### Intermittent Use Is Preferred

An intermittent use of CGMs was expressed as the preferred way to use CGMs for many participants. It was, however, reported as crucial to have access to CGMs during periods of perceived or unmet needs. Some commented that access to CGMs in the initial phase after diagnosis would have abled them to make necessary changes in their lives at an earlier stage. The importance of this is illustrated by the following quote:

If I had a CGM several years ago, when I got diabetes, I am convinced that I would not be in the situation that I am now. I think it would have helped me to understand the effect of exercise and food. . . . It would have empowered me and improved my health. (Inf 12, first int)

The participants expressed that traditional finger-pricking did not give them the same understanding, security, and overview of the glucose levels as CGMs do. One said:

It is not possible to compare [CGM to SMBG]. Because to get the same richness of information and understanding of the disease with the traditional way of measuring blood glucose, you would have to measure so often that . . . well, then the focus on diabetes would have been the only thing in your life. (Inf 14, first int)

Finger-pricking was described as burdensome and could induce a feeling of guilt and self-blame when they did not measure as often as they thought they should. To measure glucose by finger-prick was also expressed as “old-fashioned,” and bringing along the necessary equipment for SMBG was often not prioritized.

Intermittent, as compared to continuous, use of CGM was highlighted, especially in the second interviews. In these interviews, the initial enthusiasm had declined a bit. The participants in the follow-up interviews had a fairly uniform opinion that there was no need for intensified follow-up of glucose levels with CGMs during periods with satisfactory A1C. Regardless, CGMs were described as an appropriate support in diabetes self-management, especially during challenging periods, such as illness, vacations, and other situations that could easily disrupt the individual’s rhythm or control. One expressed it like this:

When things in the body are not stable, things that can occur in life, like a depression or if something happens . . . for example vacations could be such a situation. When you eat and drink stuff that you are not used to, like alcohol and such. (Inf 1, second int)

During the follow-up interviews, 2 years after the first time on a CGM, some described that they used CGMs periodically even though they had to pay for it themselves. They considered the costs as an investment in the future, with the aim of preventing diabetes-related complications. One said:

I have chosen to buy some CGMs myself because it is so valuable for my self-management. . . . But I think that paying so much to get it is a little unfair. You get help to finance medicine, but not to equipment that can entail less use of medicine. (Inf 12, second int)

### A Balancing Act

During the first period with CGMs, some participants described an urge to check the glucose level “unnecessarily often” because they were curious and experienced it as exciting and fun to be able to observe the glucose level so easily. However, some drawbacks and stress related to CGMs were also described. One participant said:

It was a period of testing [in the beginning], and I was awakened by alarms during the night, because I didn’t have any control over my glucose levels [at that time]. (Inf 1, first int)

Another commented:

Even though I know that the glucose level is at its highest after food intake, to see the spikes is a little stressing . . . , and I checked at night, and that made me anxious, so it was a little stressful. (Inf 6, first int)

In addition, practical issues, such as inserting the sensor, trusting the accuracy of the reported glucose levels, and the possibility that it could come loose, were mentioned as drawbacks. One expressed an experience of being “watched over” or “controlled” by the CGM. Thus, the immediate results from the CGM could lead to unwanted feelings of pressure and guilt. One said:

Because . . . you constantly find yourself in situations where you have to resist temptations, but it can be too much with all the micro-choices you have to take, and it can lead to a guilty conscience. (Inf 10, second int)

Overall, the participants with T2DM in this study highlighted the importance of taking individual needs into account when deciding how people with T2DM should measure their glucose level. To have the opportunity to use CGMs intermittently was emphasized as extremely valuable.

## Discussion

The purpose of this study was to explore experiences with use of CGMs in people with T2DM who participated in an interdisciplinary concentrated group intervention. The main findings were that the participants with T2DM experienced the use of CGMs as a revolution, leading to enhanced understanding of how insulin works in the body and how their body reacts to various actions related to food intake and activity. In combination with the micro-choice-based concentrated group intervention, the device facilitated understanding and learning. The participants underscored the critical importance of addressing individual needs related to glucose measurement strategies, emphasizing the value of having at least intermittent access to CGMs. Traditional SMBG was considered as troublesome and old-fashioned compared to CGMs. However, a degree of stress related to CGM use was also expressed, emphasizing the need for an individual approach in the use of CGMs for T2DM management.

It has previously been shown that use of CGMs in T2DM may lead to direct insight and understanding of what affects the function of insulin and thus influences behavior and actions in daily life.^29,30^ In this study, the same was described by the participants. CGMs in combination with information and education gave novel insights into how the action of insulin can be influenced by the individual’s choices and actions, thus promoting useful actions to achieve improved diabetes self-management. Traditional SMBG would require an extensive number of measurements through finger-pricking to achieve comparable results in terms of understanding and insight. Structured and intensified SMBG has, however, been shown to improve glucose variability for noninsulin-treated T2DM.^12^ Nonetheless, the incidence of people with T2DM having a well-controlled diabetes has not increased as expected in recent years, one reason perhaps being limited access to CGMs and inadequate education for people about the importance of how to achieve satisfactory glycemic control.^2^ Findings in this study suggest that intermittent use of CGMs can be a beneficial tool in DSME, and in line with previous studies, the findings indicate that CGMs can entail an increased understanding of blood glucose fluctuations and subsequently increase patient activation and self-management.^30,31^ Thus, CGMs together with DSME may positively impact people’s health literacy.

As for the concentrated intervention, the importance of the many daily micro-choices became clearer for the participants when they received the immediate feedback from the CGM. This offered an opportunity to immediately evaluate the consequences of their micro-choices, for example, related to food intake. The same was revealed in connection with physical activity. The opportunity to see the impact of how activity, for example, a walk, increased insulin sensitivity and hence optimized glucose levels. Furthermore, CGMs can lead to increased understanding of when and why a walk can be beneficiary, an understanding beyond the fact that physical activity is generally healthy. This type of understanding is almost impossible to achieve without the biofeedback provided through visual numbers and graphs at immediate access, and this may argue for the worthy utilization of CGMs in educational purposes to improve self-management.^29^

In this study, the participants highlighted the value of having access to CGMs in situations where the daily routines were disrupted. The use of CGMs intermittently can also give individuals the possibility to relearn knowledge concerning the processes in the body as the condition progresses. It can thus be argued, as also suggested by others,^32,33^ that people with T2DM should have the opportunity to use CGMs intermittently, giving them a chance to make appropriate treatment changes and adjustments to their progressive T2DM.

The cost associated with CGMs is often presented as a barrier to offering the device to people with T2DM.^33^ National health care systems have different approaches, but most often, CGMs are not offered to people with T2DM, leaving individuals to bear the financial burden of using the device. However, the novel approach of intermittent use would not inflict the same economic burden for health care systems,^32,33^ and in a broader perspective, it may reduce the total costs related to T2DM because it may reduce complications and hospitalization.^18^ When CGMs are used intermittently, the costs may be the same or even less than intensive use of SMBG, and CGMs may lead to less overall medical expenses in the long term.^34,35^

As shown in this study, not all people with T2DM will experience use of CGM as suitable and beneficial. Individual and structured introduction to correct use of the device is important. However, some individuals may not have the capacity to understand how to use a CGM beneficially, and some might experience it as stressful and demanding. This underlines the need for an individualized approach when considering the use of CGMs in T2DM.

### Strengths and Limitations

This study provides longitudinal data and the opportunity to understand the participants’ experiences over time. By interviewing the same participants twice, a deeper and more nuanced understanding of the intervention’s impact was gained, capturing immediate and longer-term experiences. However, the second interviews consisted of only nine participants which could be a limitation. On the other hand, the interviews were considered by the analysis team to be of sufficient information power and richness to be included in the final interpretation. The trustworthiness of the study was increased by strengthening credibility, dependability, confirmability, and transferability. A research team with extensive expertise in diabetes, the use of CGMs, and qualitative research methods strengthened the study’s credibility. However, having a preunderstanding of the phenomenon under investigation can be both a strength and a limitation. On the positive side, preunderstanding can help researchers in constructing a relevant interview guide and ask adequate follow-up questions during the interviews, thereby yielding rich and thorough data. Conversely, the researchers` preunderstanding can lead to a shallow analysis. To avoid the latter and to strengthen dependability, a reflective, open-minded discussion of the data took place during the analyses. In addition, direct quotations were used to illustrate different perceptions. Confirmability of the study was maintained through a well-described design, strategic selection aiming to reach a rich variety of perceptions, and an interdisciplinary research team. The rich variety of perceptions supports the study’s transferability, although further perceptions possibly may be found in other similar settings. Nonetheless, the detailed descriptions of the context, setting, and participant characteristics strengthen the potential for transferability.^36^ In addition, using the Standards for Reporting Qualitative Research checklist for reporting qualitative research strengthens the study’s validity.^37^

## Conclusion

This study indicates that CGMs are a valuable tool for gaining a deeper understanding of bodily processes, thereby enhancing patient activation and diabetes self-management for people with T2DM. Integrating CGMs with DSME was perceived as particularly beneficial because it supported more health-promoting micro-choices in everyday life. Intermittent rather than continuous use of CGMs was often preferred, and participants underscored the importance of having access to CGMs during especially challenging periods. These findings indicate that people with T2DM should be offered access to CGMs, and intermittent use may not imply greater economic burden compared to continuous use. Availability to CGMs for people with T2DM should, however, be guided by individual preferences and perceived value.
